# Complete Genome Sequence of *Sphingorhabdus* sp. YGSMI21, Exhibiting High Enantioselective Epoxide Hydrolase Activity

**DOI:** 10.1128/genomeA.01441-17

**Published:** 2018-01-18

**Authors:** Hae-Seon Kim, Sun Ho Cha, Ho Young Suk, Nyun-Ho Park, Jung-Hee Woo

**Affiliations:** a Gyeongbuk Institute for Marine Bio-Industry (GIMB), Uljin, Gyeongbuk, South Korea; b GenoTech Corporation, Daejeon, South Korea; c Department of Life Sciences, Yeungnam University, Gyeongsan, Gyeongbuk, South Korea

## Abstract

*Sphingorhabdus* sp. YGSMI21 is a novel strain exhibiting high enantioselective hydrolysis activity for styrene oxide. Here, we present its complete genome sequence, consisting of one circular chromosome (3.86 Mb) and one plasmid (0.196 Mb).

## GENOME ANNOUNCEMENT

Epoxide hydrolases (EHase) (EC 3.3.2.3) hydrolyze epoxides to their corresponding vicinal diols (1–3). EHase was recently reported as a potential biocatalyst for the production of chiral epoxides (4). In chiral compounds, it is common for biological activities to occur in one enantiomer, while other enantiomers have no activity or adverse side effects (4). Since pharmaceuticals with a chiral carbon should be developed as a single enantiomer (5, 6), there is a need for continuous study to find microbial sources of enantioselective EHase that preferably hydrolyze only one enantiomer in racemic substrates (1). EHase with a desired enantioselectivity has been screened from polycyclic aromatic hydrocarbon-degrading bacteria, resulting in the isolation of the novel strain *Sphingorhabdus* sp. YGSMI21 (7, 8). It was reported that *Sphingorhabdus* sp. YGSMI21 itself preferentially hydrolyzed the (*R*)-enantiomer of styrene oxide, yielding the (*S*)-enantiomer at greater than 99.9% efficiency (7, 8).

The extracted DNA from *Sphingorhabdus* sp. YGSMI21 was used to construct 20-kb SMRTbell template libraries. Whole-genome sequencing was conducted using the PacBio RS Ⅱ platform (Pacific Biosciences) (9), yielding 75,245 long reads totaling 770,716,593 bp after filtering of the subreads. *De novo* assembly was conducted using the Hierarchical Genome Assembly Process (HGAP) version 2.3 (10). The estimated genome size was 4,129,946 bp with an average coverage of 162×. The genome annotation was conducted using the NCBI Prokaryotic Genome Annotation Pipeline (PGAP).

The sizes of the chromosome and plasmid were 3,864,176 bp and 196,579 bp, respectively. The genome of *Sphingorhabdus* sp. YGSMI21 contained 3,993 protein-coding genes, 47 tRNAs, and 6 rRNAs with a G+C content of 56.0%. Our analysis confirmed the presence of three epoxide hydrolases and one limonene-1,2-epoxide hydrolase in this genome.

### Accession number(s).

The complete genome sequence of *Sphingorhabdus* sp. strain YGSMI21 was deposited at GenBank under the accession numbers CP022548 and CP022549. *Sphingorhabdus* sp. YGSMI21 is currently available from the Korean Culture Center of Microorganisms with the accession number KCCM 12136P.

## References

[1] Weijers CAGM, de Bont JAM 1999 Epoxide hydrolases from yeasts and other sources: versatile tools in biocatalysis. J Mol Catal B Enzym 6:199–214. doi:10.1016/S1381-1177(98)00123-4.

[2] Kang JH, Woo JH, Kang SG, Hwang YO, Kim SJ 2008 A cold-adapted epoxide hydrolase from a strict marine bacterium, *Sphingophyxis alaskensis*. J Microbiol Biotechnol 18:1445–1452. 18756107

[3] Saini P, Sareen D 2017 An overview on the enhancement of enantioselectivity and stability of microbial epoxide hydrolases. Mol Biotechnol 59:98–116. doi:10.1007/s12033-017-9996-8. 28271340

[4] Lee EY, Shuler ML 2007 Molecular engineering of epoxide hydrolase and its application to asymmetric and enantio-convergent hydrolysis. Biotechnol Bioeng 98:318–327. doi:10.1002/bit.21444. 17405175

[5] Nguyen LA, He H, Pham-Huy C 2006 Chiral drugs: an overview. Int J Biol Med Sci 2:85–100. PMC3614593 23674971

[6] Yu HL, Xu JH, Lu WY, Lin GQ 2009 Discovery and utilization of biocatalysts for chiral synthesis: an overview of Chinese scientists’ research and development. Adv Biochem Eng Biotechnol 113:1–31. doi:10.1007/10_2008_30. 19623477

[7] Woo JH, Lee EY 2014 Enantioselective hydrolysis of racemic styrene oxide and its substituted derivatives using newly-isolated *Sphigopyxis* sp. exhibiting a novel epoxide hydrolase activity. Biotechnol Lett 36:357–362. doi:10.1007/s10529-013-1373-5. 24129951

[8] Woo JH, Kwon TH, Kim JT, Kim CG, Lee EY 2013 Identification and characterization of epoxide hydrolase activity of polycyclic aromatic hydrocarbon-degrading bacteria for biocatalytic resolution of racemic styrene oxide and styrene oxide derivatives. Biotechnol Lett 35:599–606. doi:10.1007/s10529-012-1114-1. 23242500

[9] Varela-Alvarez E, Andreakis N, Lago-Leston A, Pearson GA, Serrao EA, Procaccini G, Duarte CM, Marba N 2006 Genomic DNA isolation from green and brown algae (*Caulerpales* and *Fucales*) for microsatellite library construction. J Phycol 42:741–745. doi:10.1111/j.1529-8817.2006.00218.x.

[10] Chin CS, Alexander DH, Marks P, Klammer AA, Drake J, Heiner C, Clum A, Copeland A, Huddleston J, Eichler EE, Turner SW, Korlach J 2013 Nonhybrid, finished microbial genome assemblies from long-read SMRT sequencing data. Nat Methods 10:563–569. doi:10.1038/nmeth.2474. 23644548

